# Perinatal Choline Supplementation Reduces Amyloidosis and Increases Choline Acetyltransferase Expression in the Hippocampus of the APPswePS1dE9 Alzheimer's Disease Model Mice

**DOI:** 10.1371/journal.pone.0170450

**Published:** 2017-01-19

**Authors:** Tiffany J. Mellott, Olivia M. Huleatt, Bethany N. Shade, Sarah M. Pender, Yi B. Liu, Barbara E. Slack, Jan K. Blusztajn

**Affiliations:** Department of Pathology & Laboratory Medicine, Boston University School of Medicine, Boston, Massachusetts, United States of America; Nathan S Kline Institute, UNITED STATES

## Abstract

Prevention of Alzheimer's disease (AD) is a major goal of biomedical sciences. In previous studies we showed that high intake of the essential nutrient, choline, during gestation prevented age-related memory decline in a rat model. In this study we investigated the effects of a similar treatment on AD-related phenotypes in a mouse model of AD. We crossed wild type (WT) female mice with hemizygous APPswe/PS1dE9 (APP.PS1) AD model male mice and maintained the pregnant and lactating dams on a control AIN76A diet containing 1.1 g/kg of choline or a choline-supplemented (5 g/kg) diet. After weaning all offspring consumed the control diet. As compared to APP.PS1 mice reared on the control diet, the hippocampus of the perinatally choline-supplemented APP.PS1 mice exhibited: 1) altered levels of amyloid precursor protein (APP) metabolites–specifically elevated amounts of β-C-terminal fragment (β-CTF) and reduced levels of solubilized amyloid Aβ40 and Aβ42 peptides; 2) reduced number and total area of amyloid plaques; 3) preserved levels of choline acetyltransferase protein (CHAT) and insulin-like growth factor II (IGF2) and 4) absence of astrogliosis. The data suggest that dietary supplementation of choline during fetal development and early postnatal life may constitute a preventive strategy for AD.

## Introduction

The development of a treatment for AD constitutes a major goal for biomedical sciences. A vast amount of resources have been devoted to this challenge, consistent with its enormous societal need. Very little thought has been given however to the possibility that AD might be preventable or that its onset might be delayed by the use of a prevention strategy. We have previously shown that high choline intake during gestation and perinatal period in rodent models prevents age-related memory decline [1] and in the current study we test the idea that this preventive strategy will be effective in a model of AD. Choline was classified as an essential nutrient, by the Food and Nutrition Board (FNB) of the Institute of Medicine of the National Academy of Sciences, relatively recently. It was only in 1998 that the FNB issued dietary reference intake values for this nutrient [2]. Significantly, the FNB recognized that the requirements for choline are increased during pregnancy and nursing (Adequate Intake values for women (mg/day): non-pregnant 425; pregnant 450; lactating 550). Because of this short history, our understanding of the significance of choline nutrition in human health and disease remains inadequate. The 2007 National Health and Nutrition Examination Survey (NHANES) study reported that in the US fewer than 15% of pregnant women consume the recommended amount [3]. Moreover, at least 25% of women in a California cohort consumed so little choline that they were at 4-fold increased risk of having babies with neural tube defects [4, 5]. Several additional studies confirm that Americans consume far less choline than recommended with only approximately 25% of adults meeting the AI values [6–10]. These data indicate that increased intake of choline by our population is a desirable public health goal.

Indeed, there is overwhelming support for this idea based on multiple studies on the effects of prenatal and early postnatal choline availability in rodents showing that high choline intake is neuroprotective in models of neuronal dysfunction, including those induced by aging [1, 11, 12], seizures [13–17], maternal alcohol consumption [18–24], Down’s syndrome [25–30], autism spectrum disorders [24, 31–36], early-life iron deficiency [37], exposure to stress *in utero* [38], and schizophrenia [39–42].

In this study, we examined the effects of perinatal choline supplementation on AD pathology in the APPswe/PS1deltaE9 (APP.PS1) mice that express murine amyloid precursor protein (APP) with the human Aβ amino acid sequence harboring mutations that cause a familial form of AD (the Swedish mutation *APP* (K595N/M596L; APPswe) and a mutated form of presenilin 1 (*PSEN1* with exon 9 deleted; PS1dE9) [43]. Although no model of AD fully recapitulates the human disease [44], APP.PS1 mice are well suited for our studies because they exhibit: 1) high production of Aβ peptides in brain and accumulation of amyloid plaques by 4–6 months of age [45], and 2) cholinergic defects [46–50]. The latter is important because a large body of evidence indicates that basal forebrain cholinergic neurons (BFCN) are vulnerable to degeneration in AD [51–55], and our previous studies in rats showed that choline supplementation *in utero* modulates acetylcholine (ACh) synthesis and release in adult BFCN [56]. Using APP.PS1 mice, we found that perinatal choline supplementation can slow the accumulation of Aβ40 and Aβ42 peptides and reduce plaque formation, which in turn may prevent the heightened gliosis found in APP.PS1 mice. The reductions in cholinergic markers, such as choline acetyltransferase (CHAT), observed in APP.PS1 mice can be rescued by perinatal choline supplementation suggesting that cholinergic function and possibly cognitive ability may be intact in these mice. Thus, dietary supplementation of choline during fetal development and early postnatal life can produce life-long changes that may protect the brain and dramatically slow the progression of AD.

## Materials and Methods

### Ethics Statement

All animal procedures were performed in accordance with the Animal Welfare Act (Animal Welfare Assurance Number A-3316-01) and the principles of the NIH Guide for the Care and Use of Laboratory Animals and were approved by the Institutional Animal Care and Use Committee of Boston University (Protocol #AN-14994).

### Animals

We used the APPswe/PS1deltaE9 (APP.PS1) mice purchased from Jackson Laboratories (strain B6C3-Tg(APPswe,PSEN1dE9)85Dbo/Mmjax, Stock #034829) [43]. Breeding pairs (APP.PS1 +/- male and WT female) were divided into 2 groups: Control and Supplemented. Unless noted, animals were maintained on a standard rodent AIN76A diet [57, 58] (Dyets #110098) consisting of 20.3% protein, 66% carbohydrate, and 5% fat. Specifically, this diet contained (per kg) Casein (200 g), DL-methionine (3 g), corn starch (150 g), sucrose (500 g), cellulose (50 g), Corn oil (50 g), mineral mix S10001 (35 g), and vitamin mix V10001 (10 g). From the time of mating until offspring were weaned, dams were given either a control AIN76A diet (Dyets #110098) containing 1100 mg/kg of choline chloride or a choline-supplemented diet (Dyets #110184) containing 5000 mg/kg. After weaning at postnatal day (P) 21, all offspring were fed a control diet. All experiments were performed using the transgenic and non-transgenic (control) littermates. Mice were euthanized at 6-, 9-, and 12-months of age. The number of animals (N) per age, sex and group were as follows: 6-months (females: control WT N = 6, control APP.PS1 N = 4, supplemented WT N = 4, and supplemented APP.PS1 N = 4; males: control WT N = 4, control APP.PS1 N = 5, supplemented WT N = 4, and supplemented APP.PS1 N = 5), 9-months (females: control WT N = 5, control APP.PS1 N = 6, supplemented WT N = 5, and supplemented APP.PS1 N = 5; males: control WT N = 6, control APP.PS1 N = 7, supplemented WT N = 6, and supplemented APP.PS1 N = 6), and 12-months (females: control WT N = 6, control APP.PS1 N = 7, supplemented WT N = 6, and supplemented APP.PS1 N = 7; males: control WT N = 7, control APP.PS1 N = 8, supplemented WT N = 6, and supplemented APP.PS1 N = 4). Samples from all animals were analyzed in all assays described below.

At each time point, mice were euthanized with CO_2_, and decapitated. Brains were rapidly removed. One hemisphere was immediately fixed for tissue staining and the other was dissected on ice. The hippocampus was used for protein analysis.

During the study 10 animals died and were not used for experimental purposes: 1 was a control diet WT female (unknown cause/found dead), 2 were control diet APP.PS1 females (both were humanely euthanized due to poor body condition), 6 were control diet APP.PS1 males (4 were found dead in their cages, 2 were humanly euthanized due to severe fight wounds and poor body condtion), and 1 was a choline supplemented APP.PS1 female (humanely euthanized due to poor body condition).

### ELISA for Solubilized Aβ Levels

Whole hippocampi were snap frozen on dry ice and stored at -70°C until use. Frozen tissues were sonicated in lysis buffer (0.05 M Tris-HCl pH 7.5, 0.15 M NaCl, 1% NP-40, 1 mM Na-orthovanadate, 0.001% sodium fluoride, 1% protease inhibitor cocktail (Sigma)) and centrifuged to clear. The supernatants were transferred to new tubes and stored at -70°C. A solution of 8.2 M guanidine / 82 mM Tris HCl (pH 8.0) was added to the extracts to yield a solution with 5 M final guanidine concentration. Samples were diluted with 10x volume of PBS and centrifuged at 16,000 x g for 20 minutes at 4°C. The supernatant was carefully collected and stored on ice until analyses with the Aβ40 or Aβ42 ELISA kit from Invitrogen. ELISAs were performed according to manufacturer’s instructions (Invitrogen #KHB3482 and #KHB3442, respectively).

### Western Blot Analysis

Hippocampal extracts were prepared by sonicating in lysis buffer and centrifuged to clear as described above for ELISAs. The supernatants were transferred to new tubes and stored at -70°C. The extracts were normalized for total protein and 40 μg of hippocampal protein per sample was subjected to SDS-PAGE using 4–12% Bis-Tris Midi gels (Invitrogen). After transferring to a nitrocellulose or PVDF membrane using an iBLOT apparatus (Invitrogen), the membrane was blocked with 5% nonfat dry milk in 1X TBS containing 0.1% Tween-20 for 1 h and then was probed with primary antibody overnight. The antibodies used included a monoclonal β-actin antibody (Sigma #A5441; 1:5000), a monoclonal APP antibody clone 6E10 (BioLegend #SIG-39320; 1:1000), a polyclonal C-terminal APP antibody (Calbiochem #171610, 1:1000), a polyclonal CHAT antibody (Millipore #AB144P; 1:750), a polyclonal doublecortin (DCX) antibody (Cell Signaling Technologies #4604; 1:1000), a monoclonal glial fibrillary acidic protein (GFAP) antibody (Cell Signaling Technologies #3670; 1:1000), and a monoclonal insulin-like growth factor II (IGF2) antibody (Upstate #05–166; 1:500). The antibody/antigen complexes were detected with either anti-rabbit, anti-mouse or anti-goat IgG peroxidase conjugates and visualized using the enhanced chemiluminescence method (SuperSignal West Femto Substrate, Thermo Scientific) and Kodak ImageStation 440 and quantified with the Kodak 1D software. The membranes were stripped in Restore Western Blot Stripping Buffer (Thermo Scientific) for 30 min at 37°C. After incubation with 5% nonfat dry milk in 1X TBS containing 0.1% Tween 20 for 1 h, membranes were reprobed with primary antibody as above. Densitometric values for each protein were normalized to β-actin values.

### Immunohistochemistry

Brains were dissected and immediately fixed in 10 volumes of PLP fixative (4% paraformaldehyde, 75 mM lysine, 10 mM sodium periodate; pH 7.4) at 4°C for 24 h, then cryoprotected in a graded series of 10% and 20% glycerol/2% dimethylsulfoxide, in 0.1 M PBS, pH 7.3 (24 h each). Serial, frozen sections (40 μm, coronal) were cut from the anterior frontal pole to the caudal occipital region with a sliding microtome. For Aβ40 and Aβ42 immunohistochemistry, sections were washed for 10 min in PBS and then transferred to > 95% Formic Acid for 2 min with gentle agitation. The sections were blocked in PBS/10% goat serum for 1 hour at room temperature. Sections were probed with rabbit anti-Aβ40 (Invitrogen #44–344; 1:2500) or rabbit anti-Aβ42 (Invitrogen #44–344; 1:2500) overnight at room temperature in a solution of 0.3% Triton-X 100, 2% goat serum, 0.008% sodium azide, in PBS. The next day, sections were incubated with goat anti-rabbit-HRP antibody (Millipore; 1:1000) in a solution of 2% goat serum/PBS for 3 hours at room temperature. Staining was developed in a solution containing diaminobenzidine, sodium imidazole, and hydrogen peroxide. These IHC procedures on sections slated to constitute a set used for comparative studies were performed at the same time with the same reagents under identical conditions. Mounted sections were analyzed on an Olympus B061 microscope using a 2X magnification objective, which permitted us to obtain the image of the entire hippocampus in a single photographic frame. The photographic images were obtained using constant exposure settings for each set of sections. Using the ImageJ software, the region of interest was outlined to include the entire hippocampus in each of the images. The staining intensity threshold was held constant for all of the images in a given set. Plaque number, total plaque area, average plaque size, and plaque burden were measured by the ImageJ software. Three sections per animal of the anterior (bregma approximately -1.5 mm) and posterior (bregma approximately -3 mm) hippocampus were used, and the data averaged to obtain a single value for either anterior or posterior hippocampus of that animal. Then the data were used to calculate the mean and standard error for each group of animals per region. The analysis was performed by a single individual (OMH) and subsequently verified by another person (TJM), both of whom were blinded to the identity of the samples (dietary group, age, and sex status).

### Immunofluorescence Imaging

Serial, frozen sections (40 μm, coronal) were prepared as described above. For immunofluorescence staining of DCX and GFAP, free floating sections were incubated for 3 h in a blocking buffer consisting of 10% normal donkey serum and 0.3% Triton X-100 in PBS and subsequently overnight in 1% BSA, 0.3% Triton X-100 in PBS containing either a goat anti-DCX (Santa Cruz #SC8066; 1:250) or a rabbit anti-GFAP (Sigma #180063; 1:1000), respectively. After rinsing with PBS, the sections were blocked in the aforementioned blocking buffer for 3 h and incubated in the dark for 6 h with either secondary Alexa Fluor-594 donkey anti-goat IgG antibody (Life Technologies; 1:1000) or secondary Alexa Fluor-594 donkey anti-rabbit IgG antibody (Life Technologies; 1:1000). The sections were then rinsed in PBS. After the final PBS rinse, the sections were mounted on SuperfrostPlus slides (Fisher), allowed to dry at RT in the dark, coverslipped and stored at -20°C. The sections were imaged with Olympus IX81/DSU spinning disc confocal microscope.

### Data Analysis

Data for all experiments, presented as means ± SEM, were analyzed by t-test or a one- or two-way ANOVA, as appropriate. *Post hoc* analyses were performed with a Tukey test.

## Results and Discussion

**Fig 1:**
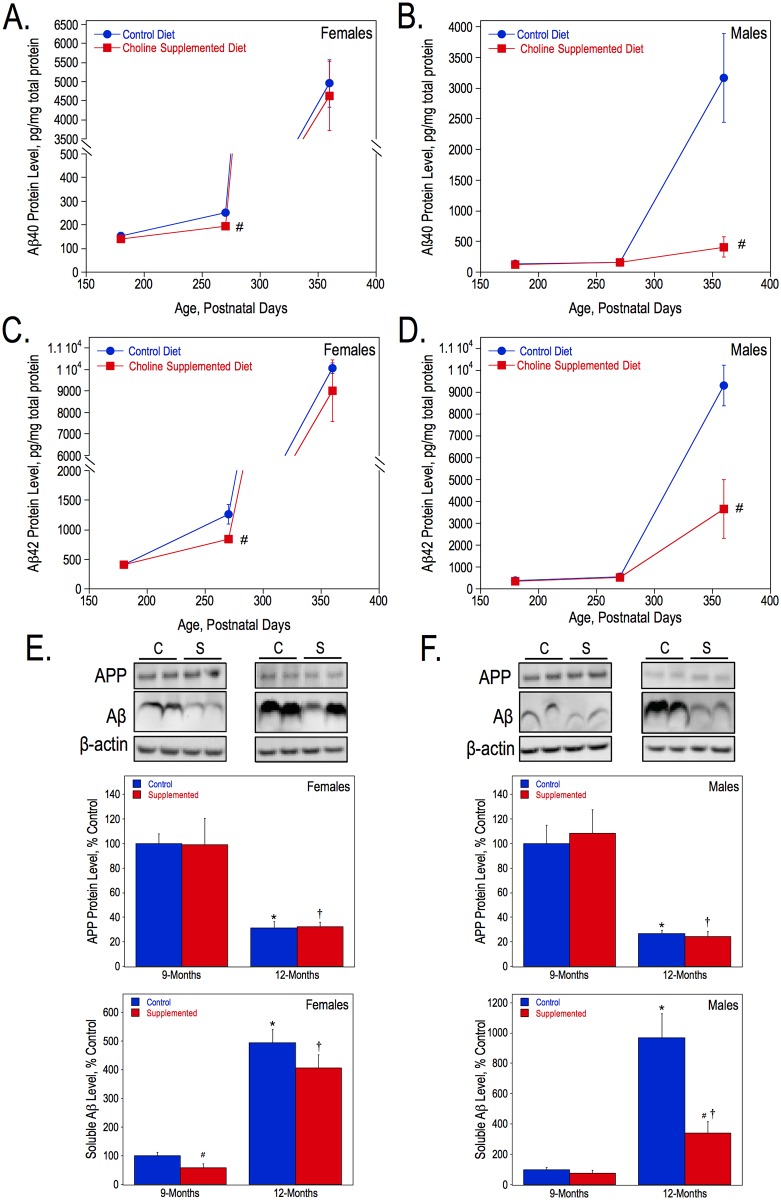
Aβ levels in the hippocampus of APP.PS1 mice. Hippocampal lysates from females and males were used to measure solubilized Aβ40 (A, B) and Aβ42 (C, D) levels by ELISA, and APP and soluble Aβ (E, F) levels by Western blot analysis using the anti-APP 6E10 antibody. For each sex, the lysates from both 9- and 12-month-old mice were loaded on the same SDS-page gel and immunoblotted together, and therefore, the data were analyzed together and presented as percentages of the 9-month control values. As determined by 2-way ANOVA for genotype and diet and Tukey test per age: * represents p<0.05 compared to control diet APP.PS1 mice at 9-months; †, p<0.05 compared to choline-supplemented diet APP.PS1 mice at 9-months; and #, p<0.05 compared to control diet APP.PS1 mice at the same age.

**Fig 2:**
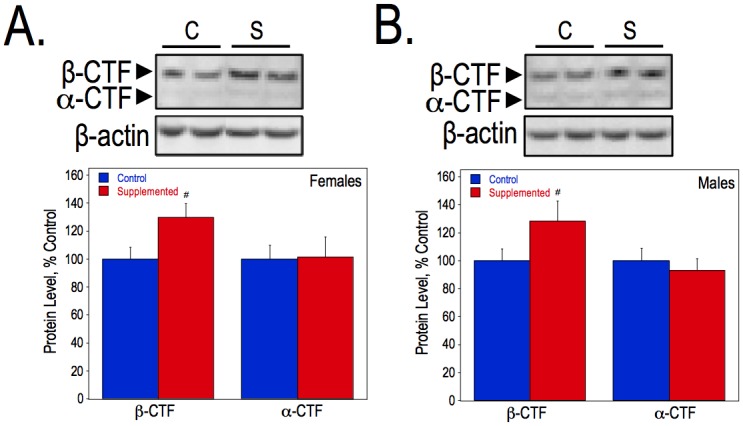
APP metabolite levels in the hippocampus of APP.PS1 mice. Hippocampal lysates from 12-month-old female and male APP.PS1 mice were used to measure the α- and β-CTFs using an anti-C-terminal APP antibody. As determined by Student T-test, * represents p<0.05 compared to control diet APP.PS1 mice of the same sex.

To assess the effects of choline supplementation on the progression of amyloidosis, we measured the amount of solubilized Aβ40 and Aβ42 by ELISA and soluble Aβ by Western blot analysis in hippocampal tissue, as well as the number of plaques and total plaque area in both anterior and posterior hippocampal sections of wild-type and APP.PS1 mice. First, solubilized Aβ40 and Aβ42 were measured in hippocampal lysates in females (Fig 1A and 1C) and males (Fig 1B and 1D) at 6-, 9-, and 12-months of age. Females from the control diet group had more solubilized Aβ40 than control males at both 9- (P270) and 12- months (P360). Choline supplementation significantly reduced the levels of Aβ40 and Aβ42 in APP.PS1 female mice at the 9-months of age but not at 12-months (Fig 1A and 1C). In contrast, there were no significant differences in Aβ40 and Aβ42 levels in 9-month-old males, but at 12-months choline-supplemented males had dramatically less solubilized Aβ40 and Aβ42 than controls (approximately 13% and 39% of controls, respectively) (Fig 1B and 1D). Western blot analysis with an anti-APP antibody was used to visualize full-length APP and Aβ levels in the hippocampus of control and choline-supplemented APP.PS1 mice. There were no significant differences in the levels of full-length human APP between dietary groups at either 9- or 12-months of age, regardless of sex (Fig 1E and 1F). In both dietary groups and in both sexes, however, there were significant reductions in the amounts of full length APP at 12-months of age compared to those at 9-months. The analysis of total soluble Aβ levels in the hippocampus via immunoblot produced similar results to those obtained using ELISA, such that choline-supplemented females had significantly less soluble Aβ than control mice at 9-months-old (Fig 1E) and choline-supplemented males had less Aβ at 12-months (Fig 1F). In both sexes, the amount of soluble Aβ significantly increased from 9-months to 12-months, regardless of diet. In the hippocampus of 12-month-old APP.PS1 mice, we also measured the levels of the products of APP cleavage catalyzed by the α and β secretase enzymes, i.e. the α- and β-C-terminal fragments (CTFs) of APP. β-CTF is the substrate of γ secretase that produces the Aβ peptides. Using Western blot analysis with an antibody raised against the C-terminal end of APP (Fig 2), we found that, while choline supplementation had no effect on the α-CTF levels, it increased the levels of the β-CTF by approximately 30% as compared to controls in both females and males (Fig 2A and 2B).

**Fig 3:**
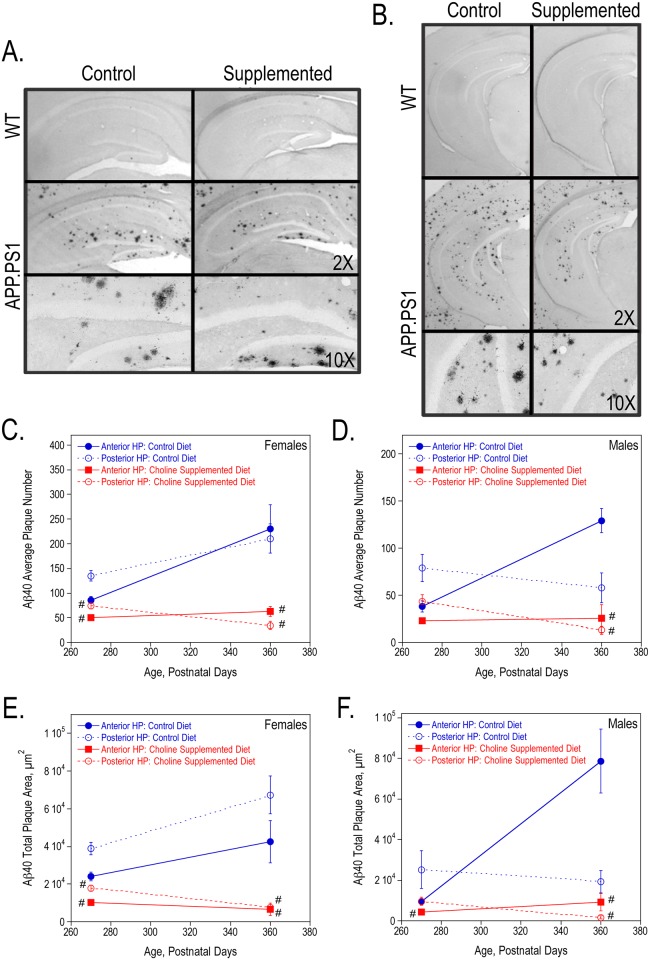
Aβ40 plaques in the hippocampus of 9-, and 12-month old WT and APP.PS1 mice. Anterior (A) and posterior (B) hippocampal sections from representative 9-month females stained with anti-Aβ40. The average number of Aβ40 plaques per animal (C, D) and the total Aβ40 plaque area (E, F) were quantified using ImageJ64 software in both females and males. As determined by 2-way ANOVA for hippocampal region and diet and Tukey test per age, # represents p<0.05 compared to control diet APP.PS1 mice at the same age. There was a significant overall effect of choline supplementation on the average number and total plaque area in both females (P270: average number p<0.001 and total plaque area p<0.0005; P360: p<0.0001 and p<0.0001) and males (P270: p<0.05 and p<0.05; P360: p<0.0005 and p<0.01).

**Fig 4:**
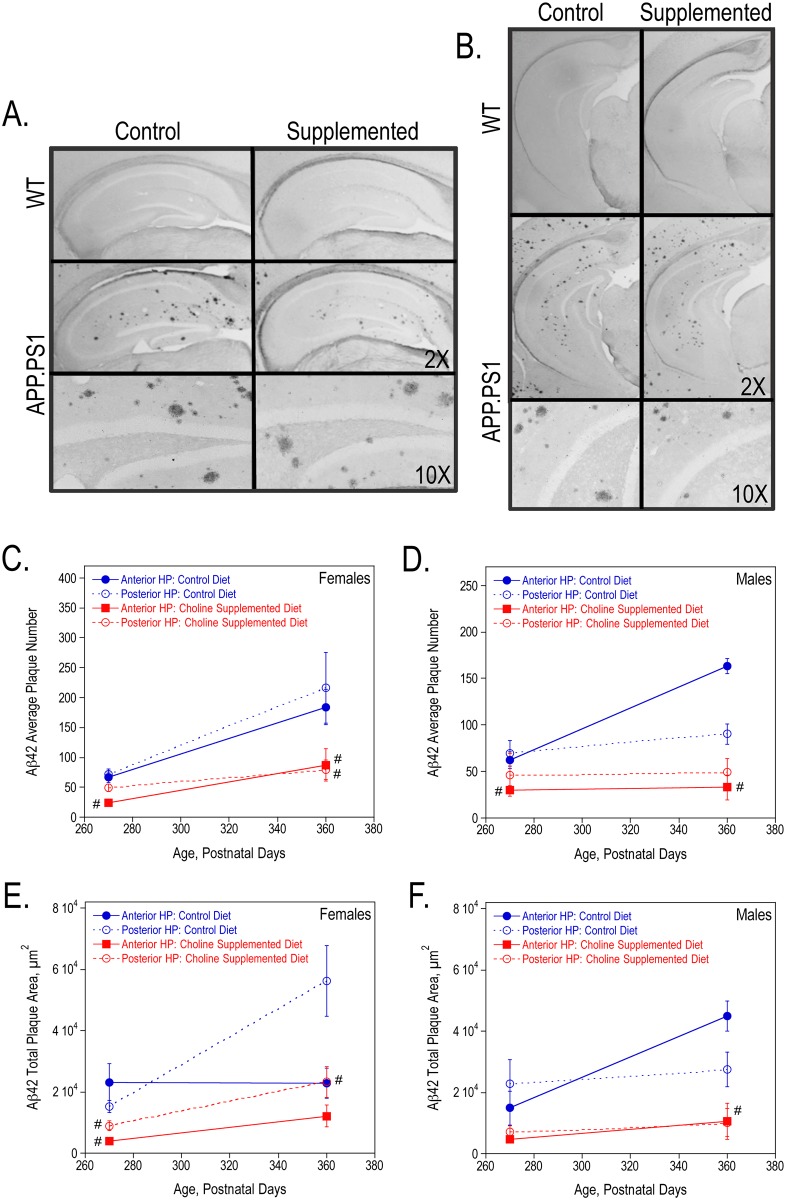
Aβ42 plaques in the hippocampus of 9-, and 12-month old WT and APP.PS1 mice. Anterior (A) and posterior (B) hippocampal sections from representative 9-month females stained with anti-Aβ42. The average number of Aβ42 plaques per animal (C, D) and the total Aβ42 plaque area (E, F) were quantified using ImageJ64 software in both females and males. As determined by 2-way ANOVA for hippocampal region and diet and Tukey test per age, # represents p<0.05 compared to control diet APP.PS1 mice at the same age. There was a significant overall effect of choline supplementation on the average number and total plaque area in both females (P270: average number p<0.005 and total plaque area p<0.01; P360: p<0.01 and p<0.05) and males (P270: p<0.05 and p<0.05; P360: p<0.005 and p<0.0005).

In addition, we used the contralateral hippocampus from these 9- and 12-month old mice to measure plaque formation using immunohistochemistry. We determined the average number, total plaque area, average plaque size and plaque burden for both Aβ40 and Aβ42 plaques in sections from the anterior and posterior hippocampus. Fig 3A and 3B show representative images of Aβ40-stained anterior and posterior hippocampal sections from 9-month old wild-type and APP.PS1 female mice. Perinatal choline supplementation significantly reduced the average number of Aβ40 plaques and total Aβ40 plaque area (also average plaque size and plaque burden- data not shown) in both 9- and 12-month old APP.PS1 females (Fig 3C and 3E) and 12-month old males (Fig 3D and 3F). Similarly, choline supplementation lowered Aβ42 plaque formation (Fig 4). Representative images of Aβ42-stained anterior and posterior hippocampal sections from 9-month old wild-type and APP.PS1 female mice are shown in Fig 4A and 4B, respectively. Choline supplementation significantly reduced the average number of plaques and total plaque area in female and male APP.PS1 mice (Fig 4C–4F). While the number and size of both Aβ40 and Aβ42 plaques increased with age in the control APP.PS1 mice, the plaque number and area were more stable in choline-supplemented mice suggesting that Aβ synthesis, clearance, and/or aggregation may be altered in these mice to prevent additional plaque formation.

**Fig 5:**
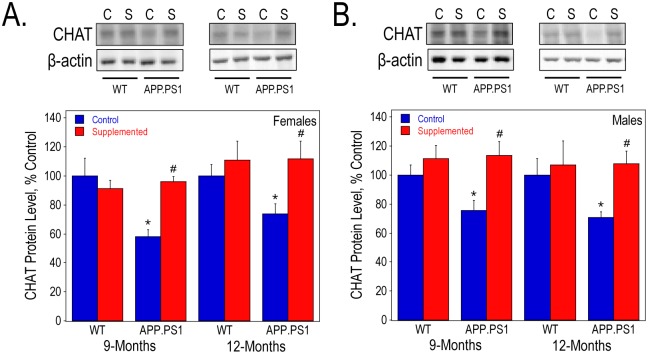
CHAT protein levels in the hippocampus of 9- and 12-month old WT and APP.PS1 mice. Hippocampal lysates were used to measure CHAT protein levels by Western blot analysis in females (A) and males (B). As determined by 2-way ANOVA for genotype and diet and Tukey test per age: * represents p<0.05 compared to control diet WT mice at the same age; and #, p<0.05 compared to control diet APP.PS1 mice at the same age.

Cholinergic dysfunction is a prominent symptom in AD and is thought to be due to the reduced expression of CHAT, the enzyme necessary for ACh synthesis, as well as the degeneration and loss of cholinergic neurons. We measured the levels of CHAT protein in the hippocampus of both control and perinatally choline-supplemented wild-type and APP.PS1 mice. At 9- and 12-months, CHAT protein levels were significantly decreased by the presence of human mutant forms of APP and PS1 in female mice from the control group (Fig 5A). Perinatal choline supplementation prevented this decrease, suggesting that choline supplementation may rescue cholinergic function in AD mice. Similar results were observed in males (Fig 5B).

**Fig 6:**
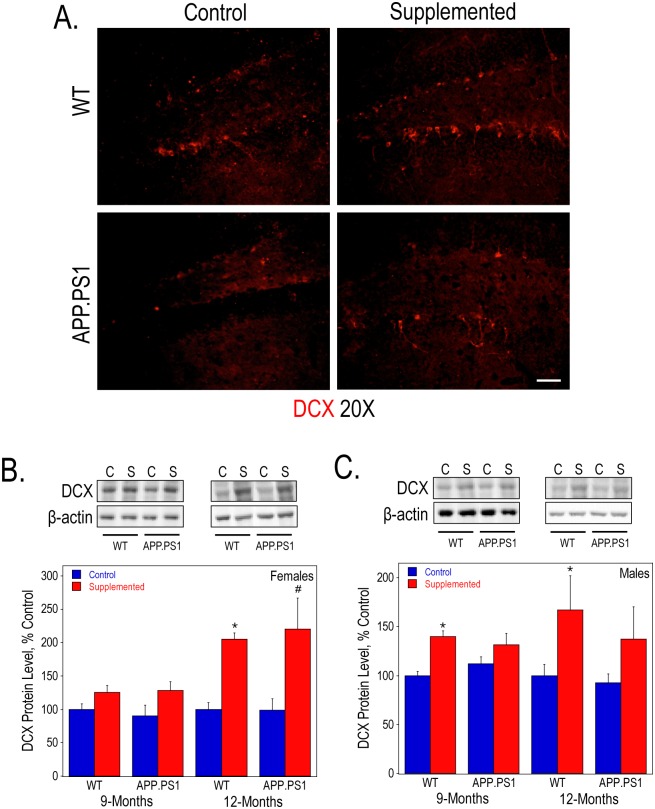
Neurogenesis in the hippocampus WT and APP.PS1 mice. DCX immunofluorescence staining of anterior hippocampal sections (A) from 9-month old female mice visualized using confocal microscopy. Bar represents 50 μm. Hippocampal lysates of 9- and 12-month old females (B) and males (C) were used to measure DCX protein levels by Western blot analysis. As determined by 2-way ANOVA for genotype and diet and Tukey test per age: * represents p<0.05 compared to control diet WT mice at the same age; and #, p<0.05 compared to control diet APP.PS1 mice at the same age. There was a significant overall effect of perinatal choline supplementation on DCX protein levels in both 9- and 12-month females (p<0.05 and p<0.0005, respectively) and males (p<0.001 and p<0.01, respectively).

We have previously shown that prenatal choline supplementation can increase hippocampal neurogenesis in rats [59, 60]. Here, we examined the effects of perinatal choline supplementation on this process in mice by immunofluorescence staining and measuring the protein expression of DCX as a marker. Overall, we did not observe any effects of the APP.PS1 genotype on DCX expression. By qualitative examination, 9-month old perinatally choline-supplemented wild-type and APP.PS1 females had more DCX-positive cells within the dentate gyrus as compared to controls (Fig 6A). Consistent with previous studies, there was an overall significant increase in DCX protein levels quantified by Western blot analysis of both female and male hippocampal lysates in the wild-type and APP.PS1 perinatally choline-supplemented mice (Fig 6B and 6C, respectively). This increase was particularly striking (over 2-fold) in 12-month old females.

**Fig 7:**
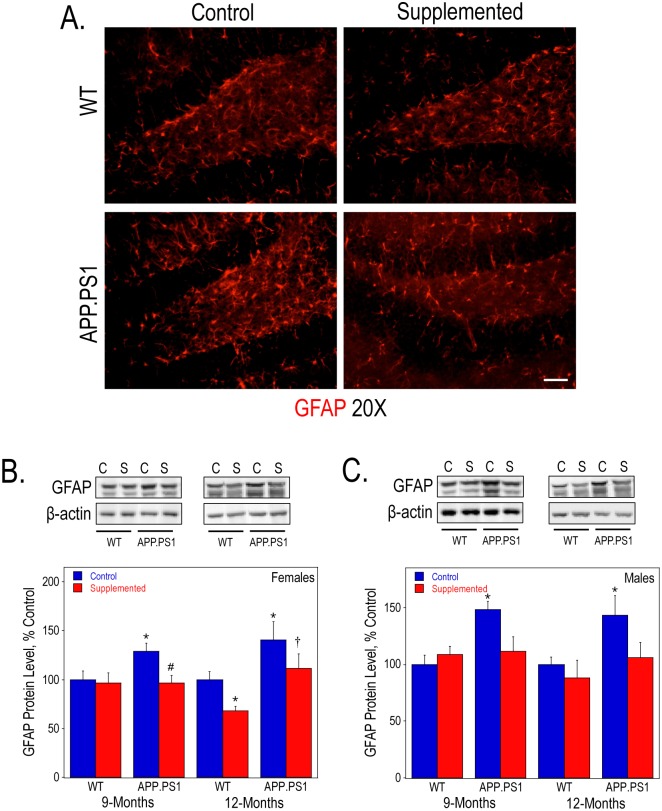
Astrogliosis levels in the hippocampus WT and APP.PS1 mice. GFAP immunofluorescence staining of anterior hippocampal sections (A) from 9-month old female mice visualized using confocal microscopy. Bar represents 50 μm. Hippocampal lysates of 9- and 12-month old females (B) and males (C) were used to measure GFAP protein levels by Western blot analysis. As determined by 2-way ANOVA for genotype and diet and Tukey test per age: * represents p<0.05 compared to control diet WT mice at the same age; †, p<0.05 compared to choline-supplemented diet WT mice at the same age; and #, p<0.05 compared to control diet APP.PS1 mice at the same age.

In addition, we determined the protein expression of GFAP as a marker for gliosis. At 9- and 12-months of age GFAP levels, as measured by Western blot analysis, were significantly increased in the hippocampus of both male and female APP.PS1 mice on a control diet (Fig 7B and 7C, respectively). This increase could be also be observed using immunofluorescence staining with an anti-GFAP antibody of hippocampal sections from 9-month old females (Fig 7A). Perinatal choline supplementation prevented the increase in GFAP expression in the APP.PS1 mice (Fig 7B and 7C). Amyloid-associated gliosis and neuroinflammation are commonly observed in the post-mortem brains of AD patients [61]. Our results in the mouse model are consistent with this observation. The decrease in GFAP protein levels in choline-supplemented mice may indicate reduced level of gliosis.

**Fig 8:**
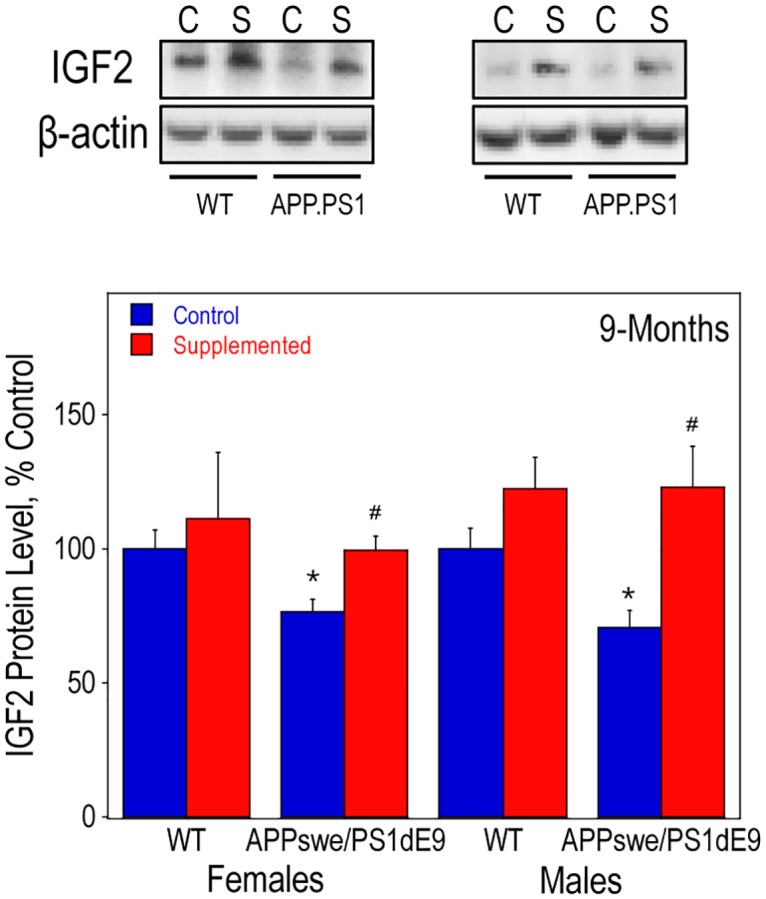
Hippocampal IGF2 protein levels in WT and APP.PS1 mice. Hippocampal lysates of 9-old females and males were used to measure IGF2 protein levels by Western blot analysis. In the males, there was a significant effect of choline supplementation, regardless of genotype, using a 2-way ANOVA (p<0.005). As determined by 2-way ANOVA for genotype and diet and Tukey test per age: * represents p<0.05 compared to control diet WT mice of the same sex; and #, p<0.05 compared to control diet APP.PS1 mice of the same sex.

Due to the emerging role of IGF2 on brain development and function, we also measured the amount of IGF2 protein in hippocampal lysates from both female and male mice at 9-months of age. Overall, choline supplementation increased hippocampal IGF2 levels (Fig 8). IGF2 levels were significantly reduced in the hippocampus of APP.PS1 mice as compared to the WT siblings; however, choline-supplemented APP.PS1 mice retained IGF2 levels similar to those of WT mice.

## Conclusions

These data show that high dietary choline consumption by mouse mothers throughout pregnancy and nursing ameliorates two central pathophysiologic features of their AD model APP.PS1 offspring: accumulation of amyloid plaques and reductions in CHAT protein levels in the hippocampus. The characteristic age-dependent progressive amyloidosis in brain of the APP.PS1 mice [45, 62] was dramatically attenuated by an increased supply of choline during fetal and early postnatal development.

Consistent with previous studies, males accumulated Aβ peptides and generated amyloid plaques more slowly than the females [63–65]. Perinatally choline-supplemented males exhibited dramatic resistance to Aβ buildup measured by ELISA and Western blot assays. However, choline-supplemented males and females showed nearly equal reductions in the number and area of Aβ40 and Aβ42 immunoreactive plaques. The latter observations suggest that while the mechanisms that control brain amyloidosis in APP.PS1 mice are sexually dimorphic, the anti-amyloidogenic mechanisms engendered by high choline supply during development operate efficiently in both sexes. Our data showing that perinatal choline supplementation increased the hippocampal levels of β-CTF in both sexes is consistent with this notion. Moreover, the pattern of reduced levels of Aβ peptides together with increased levels of β-CTF in brain of perinatally choline-supplemented animals is reminiscent of what is observed in AD mouse models treated with certain inhibitors of γ-secretase [66–68]–the enzyme that catalyzes the formation of Aβ by hydrolyzing β-CTF. Thus, the mechanism underlying amelioration of amyloidosis in our choline supplemented APP.PS1 mice may be potentially mediated by reduced activity of γ-secretase. Similarly both male and female choline-supplemented APP.PS1 mice were resistant to the 30–40% decline in the hippocampal CHAT protein levels observed in mice reared on the control diet. The vulnerability of the septohippocampal cholinergic neurons to the pathophysiologic process of AD is commonly considered to be mediated by the toxic actions of Aβ peptides [69]. Our observations are consistent with this idea, however, we note that CHAT levels reach their nadir already at 9 months–a time when accumulation of hippocampal Aβ peptides and amyloid plaques in APP.PS1 mice is far from complete. Thus, it is possible that the anticholinergic actions of Aβ are saturated at low levels of the peptide, as seen in our previous studies in cell culture [69], or that they occur in the septum in the milieu of the cholinergic neuron somata, a region generally free of amyloid plaques (data not shown), rather then the hippocampus.

AD model mice, including the APP.PS1 mice, reportedly exhibit impaired adult hippocampal neurogenesis as they age [70–73]. In this study, both the 9- and 12-month-old APP.PS1 mice had similar expression of DCX–a marker of newly-born, immature neurons [74]–as the WT mice suggesting no marked defects of neurogenesis in these mice. However, consistent with previous studies in rats [16, 59, 60] and Ts65Dn Down’s syndrome (DS) model mice [30], perinatal choline supplementation significantly upregulated dentate gyrus DCX staining and hippocampal DCX levels in both WT and APP.PS1 mice. Thus, high choline supply in early life appears to program the hippocampal neurogenic niche to support robust neurogenesis in adulthood. It remains to be determined if this effect of choline is due to its actions on the early maturation of the neuronal stem/precursor cells in the dentate gyrus *per se* or due to the modulation of the trophic environment of these cells. While there are no data on the former, the latter possibility is supported by the observations that the levels of multiple growth factors known to stimulate dentate gyrus adult neurogenesis [75] are increased in the hippocampus of perinatally choline-supplemented rats and mice. The list of such choline-responsive factors includes: NGF [15, 76], BDNF [15, 59], VEGF [60], IGF1 [15, 17], and IGF2 [77, 78].

Consistent with previous studies [79, 80], including ours [64, 81], the APP.PS1 mice were characterized by hippocampal gliosis as determined by GFAP immunofluorescence and protein level assays. This gliosis was nearly eliminated by perinatal choline supplementation. Given that activation of glial cells in AD and in AD mouse models may be initiated by Aβ peptides [61], it is possible that reduced gliosis in perinatally choline-supplemented APP.PS1 mice is secondary to the amelioration of the amyloidosis seen in these animals. However, in previous studies we observed that prenatal choline supplementation in rats similarly attenuated increases in hippocampal GFAP expression evoked by seizures [16]. Taken together the data indicate that high choline intake during development may have long-term anti-inflammatory actions in brain.

The age-associated amyloidosis of AD is the result of the accumulation of Aβ peptides that are produced by proteolytic processing of APP [82]. Similar amyloidosis is found already at a young age in the brains of patients with DS [83], caused by the inheritance of an extra copy of chromosome 21 that harbors the *APP* gene. Because the murine *App* and human *APP* genes encode proteins with somewhat different amino acid sequences, the murine Aβ peptides do not aggregate and thus produce no amyloid. For this reason, mouse models of AD (including the APP.PS1 mice) are engineered to express various forms of the human *APP*. However, models of DS have been generated (e.g. the Ts65Dn line [84]) by producing animals with an additional copy of a portion of murine chromosome 16 that is syntenic with the DS critical region on the human chromosome 21 [85]. These mice exhibit various morphological, cognitive, behavioral and brain defects that model DS [86]. Interestingly, perinatally choline-supplemented Ts65Dn mice are somewhat protected from attention and memory impairments [25, 28, 30], structural abnormalities in BFCN [25–27], and deficits of hippocampal neurogenesis [30]. These data, together with the current results, raise the possibility that some of the abnormalities seen in brains of the Ts65Dn and APP.PS1 mice may be mediated by common mechanisms related to overexpression of the amyloid precursor protein (*App* in the Ts65Dn mice and mutant *APP* in the APP.PS1 mice, respectively) or its proteolytic products [82], including the murine or human Aβ, and not necessarily by the toxic actions of the human Aβ peptides.

The overall mechanisms of action of high choline intake during fetal and early postnatal development on adult brain structure and function remain to be determined but are likely related to the metabolism of choline for use in the synthesis of membrane phospholipids (e.g. phosphatidylcholine) and as a precursor of ACh. Moreover, following enzymatic oxidation to betaine, choline functions as a methyl group donor and as such influences DNA and histone methylation–two central epigenomic processes that regulate gene expression [87]. Indeed, we [88, 89], and others [90–93] have shown that perinatal availability of choline dramatically alters brain DNA and histone H3 methylation. In an earlier set of studies we found that prenatal choline intake modulates the methylation patterns of the regulatory DNA elements in the gene encoding IGF2 [88], and that IGF2 mRNA and protein levels are dramatically upregulated by prenatal choline supplementation in the hippocampus and cerebral cortex of rats [77, 78]. IGF2 is highly expressed in the choroid plexus and secreted into the CSF [94, 95], and thus may exert global influence on the brain. Previous studies showed that intrahippocampal injections of IGF2 in young rats [96–98] and mice [99, 100] enhances memory function, whereas antagonizing the action of endogenous IGF2 impairs memory [96, 99, 101] indicating the possible role of brain-derived IGF2 in this process. IGF2 upregulates the proliferation of neural stem cells in the dentate gyrus [102], and intrahippocampal injections of IGF2 promote the survival of adult-born neurons in the dentate granule cell layer [99, 100]. In addition, we [78], and others [103] found that IGF2 increases the release of ACh from BFCN. Most importantly, we reported that intracerebroventricular IGF2 infusion ameliorates the amyloidosis and the cholinergic defect in the APP.PS1 mice [81]. In this study, choline supplementation increased IGF2 levels and prevented the reduction in IGF2 protein observed in APP.PS1 mice. Thus, it is possible that many of the actions of high choline intake observed in this study are mediated by IGF2.

Our study used a mouse model of AD that causes severe AD-like pathology due to the overexpression of mutant *APP* and *PSEN1* genes that cause hereditary forms of the disease in humans. We found that the severity of the AD-like symptoms in this model can be significantly attenuated by the supplementation of maternal diet with choline during pregnancy and nursing. The vast majority of human AD is sporadic with no known causes and even though its prevalence is alarming, reaching over 30% in individuals over 85 years of age [104], the disease does not appear to be an inevitable result of aging. Some of the factors that prevent or forestall AD have may be genetic; e.g. non-carriers of the *APOE* ε4 allele [105–108] or individuals who inherited the rare APP *A673T* allele [109] may be somewhat protected. Our study suggests that vulnerability to AD may be modified by early-life nutrition and further support the notion that adequate intake of choline during pregnancy and nursing in an important public health goal.
