# Correction: Perinatal Choline Supplementation Reduces Amyloidosis and Increases Choline Acetyltransferase Expression in the Hippocampus of the APPswePS1dE9 Alzheimer's Disease Model Mice

**DOI:** 10.1371/journal.pone.0174875

**Published:** 2017-03-23

**Authors:** Tiffany J. Mellott, Olivia M. Huleatt, Bethany N. Shade, Sarah M. Pender, Yi B. Liu, Barbara E. Slack, Jan K. Blusztajn

The Data Availability statement for this paper is incorrect. The correct statement is: Data are available from the figshare repository: (https://doi.org/10.6084/m9.figshare.4728631.v1).
